# Neuroinflammation and acquired traumatic CNS injury: a mini review

**DOI:** 10.3389/fneur.2024.1334847

**Published:** 2024-02-21

**Authors:** Michelle H. Theus

**Affiliations:** ^1^Department of Biomedical Sciences and Pathobiology, Virginia Tech, Blacksburg, VA, United States; ^2^Center for Engineered Health, Virginia Tech, Blacksburg, VA, United States

**Keywords:** inflammation, immunometabolism, glia, traumatic brain injury, spinal cord injury, Neurotoxic injury, phenotypic plasticity, peripheral immune cells

## Abstract

Acquired traumatic central nervous system (CNS) injuries, including traumatic brain injury (TBI) and spinal cord injury (SCI), are devastating conditions with limited treatment options. Neuroinflammation plays a pivotal role in secondary damage, making it a prime target for therapeutic intervention. Emerging therapeutic strategies are designed to modulate the inflammatory response, ultimately promoting neuroprotection and neuroregeneration. The use of anti-inflammatory agents has yielded limited support in improving outcomes in patients, creating a critical need to re-envision novel approaches to both quell deleterious inflammatory processes and upend the progressive cycle of neurotoxic inflammation. This demands a comprehensive exploration of individual, age, and sex differences, including the use of advanced imaging techniques, multi-omic profiling, and the expansion of translational studies from rodents to humans. Moreover, a holistic approach that combines pharmacological intervention with multidisciplinary neurorehabilitation is crucial and must include both acute and long-term care for the physical, cognitive, and emotional aspects of recovery. Ongoing research into neuroinflammatory biomarkers could revolutionize our ability to predict, diagnose, and monitor the inflammatory response in real time, allowing for timely adjustments in treatment regimens and facilitating a more precise evaluation of therapeutic efficacy. The management of neuroinflammation in acquired traumatic CNS injuries necessitates a paradigm shift in our approach that includes combining multiple therapeutic modalities and fostering a more comprehensive understanding of the intricate neuroinflammatory processes at play.

## Graphical abstract

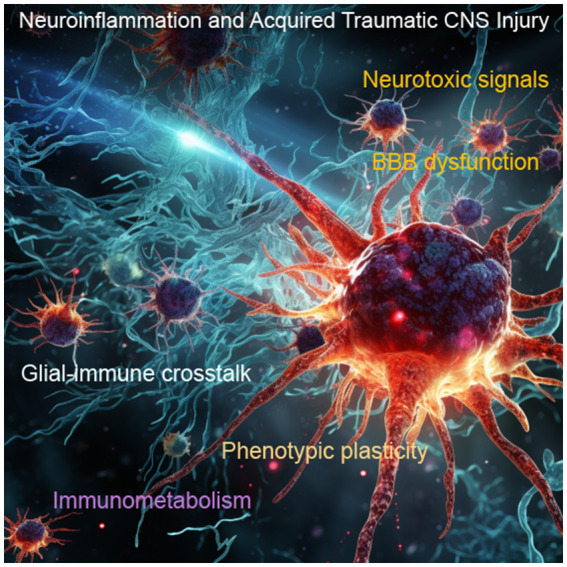

## Introduction

Neurotrauma research has undergone a remarkable transformation in recent years. One of the most pressing and intriguing areas of study is the intricate nexus between neuroinflammation and traumatic injuries to the central nervous system (CNS). Traumatic brain injury (TBI) and spinal cord injury (SCI), pose significant challenges to both patients and healthcare providers. These injuries result from a wide range of traumatic events, including accidents, falls, sports-related incidents, and acts of violence, making them a global public health concern (1, 2). Understanding the etiology of TBI and SCI is essential to appreciate the complexity of these conditions and the hurdles they present to effective treatment. Mechanisms of injury include the primary initial impact and the direct damage it inflicts on neural tissue, as well as secondary injury, which evolves over hours to days due to complex pathological processes (3, 4). Secondary injury mechanisms include neuroinflammation, oxidative stress, and excitotoxicity, all of which exacerbate tissue damage and hinder recovery (5, 6). The severity of TBI can range from mild, often characterized by temporary cognitive impairment, to severe, causing profound and lasting neurological deficits (7, 8). The heterogeneity of TBI’s etiology and outcomes underscores the need for individualized treatment approaches. Moreover, recent emphasis has been placed on mechanistic endophenotypes of TBI, which includes neuroinflammation, that may aid in biomarker-based improvements in diagnostic and prognostic trajectories (9). Likewise, the location and severity of SCI are key determinants of the functional deficits experienced by patients. Currently, no curative treatment exists for SCI, making it a life-altering condition with limited therapeutic options.

The role of neuroinflammation in the pathophysiology of traumatic CNS injury is a subject of increasing importance. Neuroinflammation plays a pivotal role in both the acute and chronic phases of these injuries, with the potential to either exacerbate damage or contribute to recovery (10). Neuroinflammation is a dynamic and complex response involving the activation of glial cells, infiltration of immune cells, and the release of various inflammatory mediators within the injured CNS (11). This response is triggered as a protective mechanism to clear debris, fight infection, and facilitate tissue repair. In the acute phase of CNS injury, neuroinflammation can exacerbate tissue damage by contributing to excitotoxicity, oxidative stress, blood–brain barrier dysfunction, and neuronal cell death. Paradoxically, it also participates in reparative processes, such as scar formation, neurogenesis, and synaptic plasticity, which can influence long-term outcomes (12). The intricate balance between neuroinflammation’s detrimental and reparative aspects remains a major challenge in the field. Elucidating the specific factors that tip the scale toward either neuroprotection or neurodegeneration is a crucial objective. Key factors such as timing, location, and duration of neuroinflammatory responses influencing acute and chronic outcomes require comprehensive investigation.

Current research seeks to decipher the intricate signaling pathways and identify potential targets for intervention, aiming to exploit the benefits of neuroinflammation while minimizing its detrimental effects. Addressing these challenges is essential for improving the prognosis and quality of life for individuals affected by traumatic CNS injuries.

## Understanding the fundamentals of neuroinflammation

Neuroinflammation is a fundamental process in the CNS characterized by the activation of immune responses within the brain and spinal cord. This complex cascade of events involves glial cells, particularly microglia and astrocytes, as well as immune cells that infiltrate the CNS (13). Neuroinflammation can have both protective and detrimental effects, serving as a double-edged sword. In its protective role, neuroinflammation helps clear debris, fight infections, and promote tissue repair (14, 15). However, when dysregulated, it can lead to prolonged inflammation, contributing to secondary damage, excitotoxicity, oxidative stress, blood–brain barrier disruption, and neuronal cell death (16).

Microglia and astrocytes play vital roles in regulating neuroinflammation, and their interactions involve complex pathways. Promoting the transition of pro-inflammatory microglia to an anti-inflammatory state, reducing the release of pro-inflammatory cytokines, and enhancing the removal of neurotoxic debris through phagocytosis is a critical component of combinatory therapy for CNS trauma (17, 18). Activated astrocytes can impact the BBB by releasing pro-inflammatory cytokines, producing reactive oxygen species (ROS), releasing matrix metalloproteinases (MMPs), upregulating glial fibrillary acidic protein (GFAP) expression, and promoting the expression of chemokines and adhesion molecules, leading to increased permeability, oxidative stress, damage to endothelial cells, and immune cell infiltration into the central nervous system (19, 20).

Microglia and astrocytes can exchange information via the release of signaling molecules such as cytokines, chemokines, and neurotrophic factors (21). For instance, microglia can release interleukin-1β (IL-1β) or tumor necrosis factor-alpha (TNF-α) in response to an inflammatory stimulus, which can then trigger astrocytes to produce chemokines like CCL2 (chemokine ligand 2), attracting immune cells to the site of injury or inflammation. Additionally, both cell types can interact through direct physical contact, such as gap junctions, which allow the exchange of ions, metabolites, and signaling molecules (22). This coordinated communication between microglia and astrocytes is essential for the regulation of neuroinflammatory responses.

## Navigating the complex interplay between the neuroimmune response

The interaction between the nervous and immune systems is a central theme in neurotrauma research with profound consequences on neurological outcomes. Microglia can adopt different activation states, ranging from a pro-inflammatory phenotype that releases cytokines and reactive oxygen species, to an anti-inflammatory phenotype that promotes tissue repair and resolution of inflammation (23). The balance between these activation states is critical in determining the trajectory of neuroinflammation and, subsequently, the extent of secondary damage and recovery. Crosstalk between neuroimmune players extends beyond microglia and infiltrating immune cells. Neurons and astrocytes actively communicate with immune cells, influencing their activation and response to injury (24–26). This includes releasing neurotransmitters and neuropeptides that can modulate the activity of microglia and peripheral immune cells.

Microglia and astrocytes also recruit neutrophils to the CNS through a coordinated release of signaling molecules, including chemokines like IL-8, CXCL1 and MIP-2, pro-inflammatory cytokines like IL-1β and TNF-α, and the upregulation of adhesion molecules like ICAM-1 and VCAM-1 on the blood vessel walls (27, 28). These mechanisms create a chemotactic and adhesive environment that guides neutrophils from the bloodstream into the CNS in response to inflammatory or injury-related cues (29). Recent efforts have begun to dissect the role of neutrophil extracellular traps (NETs), web-like structures released by neutrophils to combat infections, in adverse effects on neuronal health following trauma (30). The pro-inflammatory components within NETs trigger local inflammation, and BBB disruption, contributing to neuronal damage. Components of NETs, such as histones and proteases, can directly injure neurons, disrupt synaptic function, and induce cell death, which may lead to cognitive and motor deficits (31–33). Thus, understanding and targeting the detrimental effects of NETs in the CNS is an active area of research with the potential to offer new therapeutic avenues.

### Unraveling metabolic regulation of neuroinflammation in neurotrauma

Neuroinflammation is not a one-size-fits-all phenomenon. Its consequences can vary depending on factors such as age, sex, metabolic conditions, and epigenetic stressors (34–36). The dynamic changes in epigenetics and the reprogramming of immunometabolism influence modifications in how cells respond to internal and external signals and subsequent cell fate decisions. Metabolic regulation is a fundamental aspect of how different neuroimmune cells function within the central nervous system (CNS). Immune cells, such as microglia and infiltrating monocytes, as well as glial cells like astrocytes, adapt their metabolic profiles in response to various challenges and signaling pathways which allows for plastic use of energy substrates (37). Single-cell sequencing and omics analysis have provided valuable insights into the metabolic diversity among these cells (38, 39). Microglia, for instance, exhibit a high degree of plasticity, switching between glycolytic and oxidative phosphorylation pathways depending on their activation state. In their pro-inflammatory state, microglia tend to favor glycolysis, which generates energy quickly but is less efficient, while an anti-inflammatory state often corresponds to increased oxidative phosphorylation, which produces more ATP (37). Single-cell sequencing has unveiled the complexity of these metabolic shifts, highlighting the intricate balance required to maintain immune responses and CNS homeostasis.

Astrocytes, on the other hand, primarily rely on glycolysis for energy production (40). This metabolic profile supports their critical functions in neurotransmitter recycling and maintaining the blood–brain barrier. Importantly, astrocytes can release lactate, which serves as an energy source for neurons, emphasizing their role in the metabolic regulation of the CNS. Single-cell sequencing has revealed heterogeneity among astrocytes, suggesting that specific subpopulations may have distinct metabolic profiles and functions (41). Understanding these metabolic differences among neuroimmune and glial cells at the single-cell level is essential for developing targeted interventions that can modulate their metabolic responses, potentially optimizing neuroinflammatory processes and promoting neuroprotection in various neurological disorders.

### Diverse research approaches and developing innovative therapeutic strategies to retool the neuroimmune response

The intricacies of neurotrauma-induced neuroinflammation demand a collaborative endeavor encompassing various research methods, both in basic science and translational applications. This multidisciplinary approach fosters advances that underscore the importance of inclusivity in the research process. Current approaches to date, include the use of immunomodulatory therapies, and monoclonal antibodies that block pro-inflammatory cytokines like tumor necrosis factor-alpha (TNF) or interleukin-1β (IL-1β) that can dampen the neuroinflammatory response (42). The use of cell-based therapies, such as chimeric antigen receptor (CAR) T-cell therapy, and dendritic or myeloid-derived suppressor cell (MDSC) or mesenchymal stem cells (MSCs) therapies have been investigated (43, 44). Engineered cells may offer a controlled release of anti-inflammatory factors aimed at creating an immunosuppressive environment. Furthermore, nanotechnology-based strategies are being explored to enable targeted drug delivery to specific cell populations such as phagocytic cells (45).

Artificial intelligence (AI) and machine learning have become invaluable tools in the study of neuroinflammation due to their ability to analyze complex and vast datasets, recognize patterns, and make predictions based on data-driven insights. These technologies play a pivotal role in identifying novel biomarkers for neuroinflammatory conditions, which can aid in early diagnosis and disease monitoring (46). AI and machine learning can integrate multi-omics data, including genomics, transcriptomics, proteomics, and metabolomics, to uncover intricate molecular mechanisms underlying neuroinflammation (47). By identifying gene expression patterns, epigenetic modifications, and metabolic signatures associated with neuroinflammation, a deeper understanding of new and novel pathways may be revealed. As such, AI-powered predictive models can help assess treatment responses and prognosis, and support the categorization of neuroinflammatory endophenotypes in brain trauma (48–50). Further, neuromodulation techniques or non-invasive approaches including focused ultrasound, are being explored to directly influence the activity of neuroimmune cells within the CNS (51). These non-invasive approaches have the potential to modulate neuroinflammatory responses, by facilitating the delivery of immunotherapeutic agents into the brain and exerting direct immune-related effects (52, 53). As these technologies continue to advance, they hold the promise of revolutionizing our understanding of neuroinflammation and improving diagnostic and treatment strategies.

## Conclusion

Neuroinflammation in the context of acquired traumatic CNS injury underscores the complexity and multifaceted nature of the neuroimmune response. Recent advances in understanding the metabolic and phenotypic regulation of neuroimmune cells, the complex interplay between different cell types, and the development of innovative therapeutic strategies have shed new light on potential interventions for mitigating neuroinflammatory processes. Diverse research approaches, from single-cell omics analysis to AI integration, offer promising avenues for unraveling the intricate pathophysiological dimensions of neuroinflammation.

## Author contributions

MT: Writing – original draft.
